# Transcriptome and Metabolome Reveal Sugar and Organic Acid Accumulation in *Rosa roxburghii* Fruit

**DOI:** 10.3390/plants12173036

**Published:** 2023-08-24

**Authors:** Liyao Su, Tian Zhang, Min Wu, Yan Zhong, Zongming (Max) Cheng

**Affiliations:** State Key Laboratory of Crop Genetics and Germplasm Enhancement, College of Horticulture, Nanjing Agricultural University, Nanjing 210095, China

**Keywords:** *Rosa roxburghii*, sugar, organic acid, transcriptome, metabolome, transcription factor

## Abstract

Sugars and organic acids significantly impact fruit sensory quality, but their accumulation patterns and regulatory mechanisms during the development of *Rosa roxburghii* fruit are still unclear. We utilized transcriptomics and metabolomics to investigate genes related to sugar and organic acid metabolism in *Rosa roxburghii*. Metabolomics data revealed that sucrose, glucose and fructose were the primary sugars, whereas citric acid and malic acid were the primary organic acids in *Rosa roxburghii* fruit. We constructed the metabolic pathways of major sugars and organic acids in *Rosa roxburghii* and identified five key genes involved in sugar and organic acid synthesis. In addition, we identified a module containing 132 transcription factors that was significantly associated with sucrose, citric acid and malic acid. Based on quantitative polymerase chain reaction (qPCR), we identified 13 transcription factors involved in sugar and organic acid metabolism, including the transcription factor *RrANL2* and the sucrose synthase gene *RrSUS3*. Further yeast one-hybrid and dual luciferase assays showed that *RrANL2* could bind to the promoter of *RrSUS3* to increase its expression. These results provide new insights into the metabolism of sugars and organic acids in *Rosa roxburghii* fruit.

## 1. Introduction

Sweetness is an important factor that impacts the quality of most fruits [1]. The total amount of sugar compounds, such as sucrose, glucose and fructose, determines the sweetness of fruits, whereas differences in their relative ratios impact the unique flavor of each fruit [2,3]. For example, fructose and glucose are predominant in grapes [4], sucrose is the main sugar in watermelon [5] and muskmelon [6], and fructose is the main sugar in apples and pears [7,8]. Sucrose is one of the primary products of photosynthesis which is transported through the phloem to be stored in organs, including fruit [9]. As a key step in the sugar metabolic pathway, sucrose is converted to glucose and fructose by neutral converting enzymes. Subsequently, glucose and fructose are phosphorylated by hexokinase and fructokinase to synthesize glucose 6-phosphate (G6P) and fructose 6-phosphate (F6P). In addition, excess fructose and glucose are transported into vesicles for storage by sugar transport proteins [10].

Sourness is another factor that determines the quality and ripeness of fruit [11]. The sweet and sour taste of fruits depends primarily on the content and ratio of sugars and organic acids [12]. The accumulation of organic acids, such as malic acid, citric acid, succinic acid and tartaric acid, determines the acidity of fruits. Different kinds of acids have different acidities, which makes not only the total content of organic acids but also the relative ratios of different organic acids important for determining fruit acidity. In most fruits, malic and citric acids are the main constituents of organic acids [13]. As part of the tricarboxylic acid cycle, malic and citric acids are closely related to plant respiration and energy metabolism [14]. The impact of fruit organic acids and sugars on taste makes understanding key enzymes and regulatory factors, which impact their quantity, crucial for improving the quality of fruit and the economic value of horticultural crops.

*Rosa roxburghii* is an economically important fruit tree that is native to China, where it is mainly distributed in southwest regions such as Guizhou Province, Yunnan Province and Sichuan Province. *Rosa roxburghii* fruits have recently been found to contain very high vitamin C content (>2000 mg/100 g FW, fresh weight), in addition to other bioactive molecules such as vitamins, polyphenols, flavonoids, organic acids and Superoxide dismutase [15,16]. Despite these beneficial nutritional qualities, *Rosa roxburghii* fruit has a slightly sour and bitter taste, reducing its potential consumer appeal. At present, *Rosa roxburghii* fruit is primarily utilized in an array of processed products, including dry fruit, canned fruit and fruit drinks [15]. To improve the value of fresh *Rosa roxburghii* fruit, a better understanding of the dynamic changes of sugar and organic acid content during the course of its development is required. Although the metabolic processes associated with sugars and organic acids are well known in many plant species, they are poorly understood during *Rosa roxburghii* fruit development. A better understanding of these processes can lead to the development of *Rosa roxburghii* fruit with better consumer appeal, while retaining its beneficial nutritional qualities.

In this study, the dynamics of sugars and organic acids during *Rosa roxburghii* development were analyzed through metabolomic and transcriptomic analyses. Metabolic pathways for sugars and organic acids were constructed, and several key enzymes regulating sugar and organic acid synthesis were identified. We also conducted a focused investigation of the role of transcription factors in the accumulation of sugars and organic acids in *Rosa roxburghii*. Application of weighted gene co-expression analysis (WGCNA) led to the creation of modules that correlated transcription factors with sugars and organic acids. Within the modules significantly associated with sugar and organic acid accumulation, we identified many transcription factors and selected the differentially expressed transcription factors and key enzymes regulating sugar and organic acid synthesis in *Rosa roxburghii* for quantitative polymerase chain reaction validation. The regulatory relationships of transcription factors with sugar and organic acid synthesis enzymes were also verified by yeast one-hybrid and dual luciferase assays. This study provides a reference for understanding the mechanisms of sugar and organic acid accumulation in *Rosa roxburghii* fruits which can be applied to genetic breeding and quality improvement.

## 2. Results

### 2.1. Transcriptome and Metabolome Analysis

**Figure 1:**
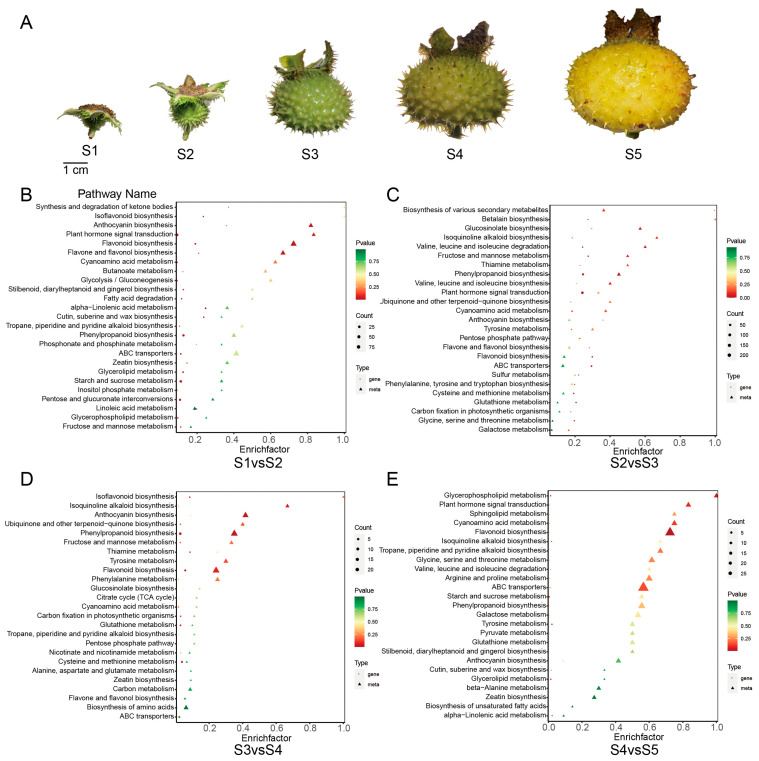
Analysis of differentially expressed genes and differentially accumulated metabolites. (**A**) The five developmental stages of *Rosa roxburghii* fruit. (**B**–**E**) KEGG enrichment of differentially accumulated metabolites and differentially expressed genes at different stages.

To study the dynamic changes of inclusion content and related genes in *Rosa roxburghii* fruits, we used transcriptomics combined with metabolomics to assay gene expression and metabolite content at five different stages of *Rosa roxburghii* fruit development (Figure 1A). Based on the OPLS-DA results, the metabolites that differed between tissues were initially identified from the obtained variable importance in projection (VIP) (VIP ≥1) of the multivariate analysis OPLS-DA model. We also combined this information with the differential gene expression data (fold change ≥ 2 and fold change ≤ 0.5) and performed KEGG analysis of differentially accumulated metabolites and differentially expressed genes in the five stages of *Rosa roxburghii* fruit development (Figure 1B–E). This analysis revealed changes in metabolites and genes associated with flavonoid biosynthesis, phenylpropanoid biosynthesis, ABC transporters, anthocyanin biosynthesis and cyanoamino acid metabolism during *Rosa roxburghii* development. Additionally, starch and sucrose metabolism, fructose and mannose metabolism, the citric acid cycle and the pyruvic acid cycle were also enriched at several time points. These results indicate that dynamic changes in sugars and organic acids take place during *Rosa roxburghii* fruit development.

### 2.2. Dynamics of Sugar and Acid Content during Fruit Development of Rosa roxburghii

**Figure 2:**
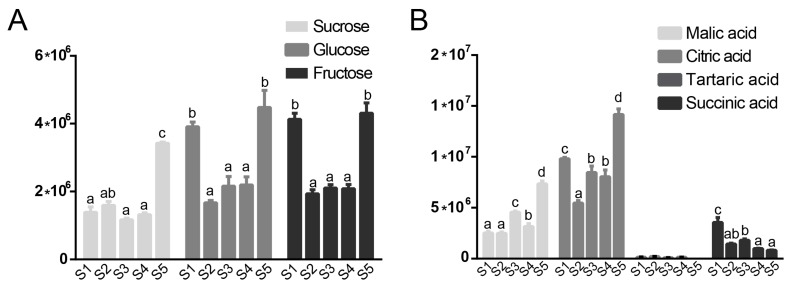
Sugar (**A**) and organic acid (**B**) content in five developmental stages of *Rosa roxburghii* fruit. a, b, c and d indicate significant difference (LSD, *p* < 0.05). Tartaric acid content was negligible compared with other acids. Therefore, we did not determine statistical significance.

Sucrose, fructose and glucose are the primary sugars present in most plant species. We found changes in the relative levels of all three sugars during the course of *Rosa roxburghii* development. The levels of these sugars decreased from the young fruit stage to the fruit expansion stage and then increased dramatically at the fruit ripening stage (Figure 2A, Table S2). For subsequent analysis, we utilized malic acid, citric acid, tartaric acid and succinic acid as representative organic acids. We found that tartaric acid content was negligible compared with other acids in *Rosa roxburghii* fruit (Figure 2B). The content of succinic acid decreased during the development of *Rosa roxburghii* fruit, reaching its lowest level in ripe fruit, indicating it is not a primary source of sour flavor (Figure 2B, Table S2). Malic acid and citric acid content, on the other hand, peaked in ripe *Rosa roxburghii* fruit. Taken together, these results demonstrate that glucose and fructose are the primary sugars in ripe *Rosa roxburghii* fruit, whereas malic acid and citric acid are the primary organic acids.

### 2.3. Metabolic Pathways of Sugars and Organic Acids in Rosa roxburghii

**Figure 3:**
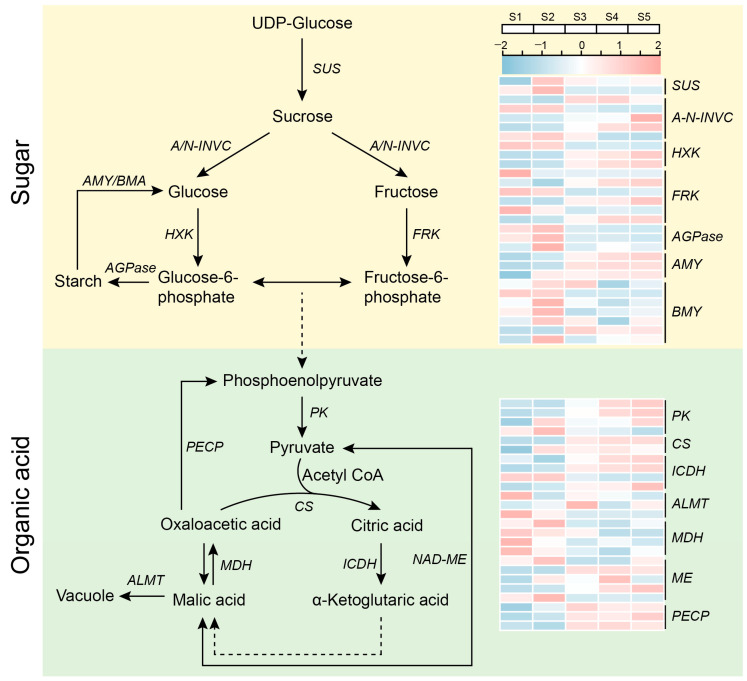
Sugar and organic acid biosynthetic pathways. SUS: sucrose synthase; A-N-INVC: invertase; HXK: hexokinase; FRK: fructokinase; AGPase: ADP-glucose pyrophosphorylase; AMY: α-amylase; BMY: β-amylase; PK: pyruvate kinase; CS: citrate synthase; ICDH: isocitrate dehydrogenase; ALMT: AL-activated malate transporter; MDH: malate dehydrogenase; ME: malic enzyme; PECP: phosphoenolpyruvate carboxylase.

The sugar and organic acid content of fruit are the two most important factors impacting its flavor. Having already identified the primary sugars and organic acids in *Rosa roxburghii* fruit, we sought to construct their metabolic pathways (Figure 3). Based on previous studies, we identified a total of 29 genes from the *Rosa roxburghii* genome that are likely involved in sugar metabolism, including members of the SUS, A-N-INVG, HXK, FRK, AGPase, AMY and BMY gene families (Table S3). SUS genes are critical for sucrose synthesis in plants, and evm.TU.chr4.1713 SUS3 expression was highest in the second fruit stage of *Rosa roxburghii*. However, another SUS gene (evm.TU.chr1.6623) was only highly expressed in S1 and S2. The A-N-INVG gene family members evm.TU.chr3.3618, evm.TU.chr5.1377 and evm.TU.chr6.3991 were highly expressed at the fruit ripening stage, and evm.TU.chr4.5684 and evm.TU.chr7.916 were highly expressed at the early stages of fruit development. In addition, members of the AGPase gene family were downregulated over the course of fruit development, reducing the conversion of sugar to starch. However, members of the AMY gene family were all upregulated during development, enhancing the degradation of starch to glucose. These findings indicate that sugar metabolism is regulated by multiple genes working together during *Rosa roxburghii* fruit development.

We identified 25 genes from seven gene families involved in organic acid metabolism in the *Rosa roxburghii* genome (Table S3). Similar to gene families related to sugar metabolism, gene families involved in organic acid metabolism also exhibited stage-specific expression profiles for different gene classes, including PK, ICDH and ME gene families. Most of these members were highly expressed at later stages of fruit development. However, the expression of MDH and ALMT gene family members was downregulated in the later stages of fruit development, whereas the expression of CS and PECP gene family members was upregulated at this same point. Analysis of these differences revealed a dynamic balance between citric acid and malic acid synthesis and metabolism in *Rosa roxburghii* fruits, with citric acid synthesis being induced later in development.

**Figure 4:**
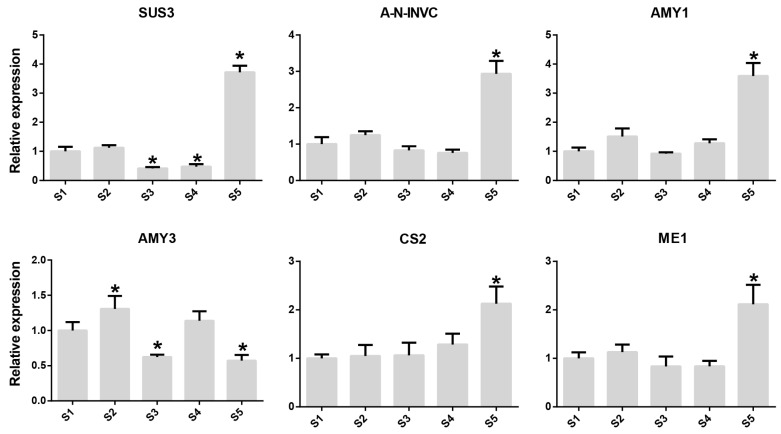
Relative expression of six genes involved in sugar and organic acid metabolism during *Rosa roxburghii* fruit development. Asterisk indicates significant difference (LSD, *p* < 0.05).

To identify the most important genes involved in sugar and organic acid anabolism in *Rosa roxburghii* fruits, we analyzed the expression profiles of *Rosa roxburghii* sugar and acid metabolic pathway gene families at different developmental stages. This analysis indicated that SUS, A-N-INVG and AMY gene family members play key roles in sugar metabolism. The expression levels of evm.TU.chr4.1713 (*SUS3*), evm.TU.chr6.3991 (*A-N-INVC*), evm.TU.chr4.5028 (*AMY1*) and evm.TU.chr3.4947 (*AMY3*) consistently increased during fruit development, with 1.58, 2.33, 7.08 and 2.45-fold increases compared with the S1 stage. There were also 4.68 and 8.61-fold increases in the organic acid metabolic genes *CS2* (evm.TU.chr1.2040) and *ME1* (evm.TU.chr1.7029), respectively. We also performed qPCR analysis on the aforementioned genes, and the results were comparable to those of RNA-seq analysis. Additionally, all surveyed genes, except *AMY3*, were significantly expressed at the S5 stage (Figure 4). These results suggest that these genes have important roles in the synthesis of sugars and organic acids in *Rosa roxburghii* fruit.

### 2.4. Co-Expression Network Construction and Transcription Factor Screening

**Figure 5:**
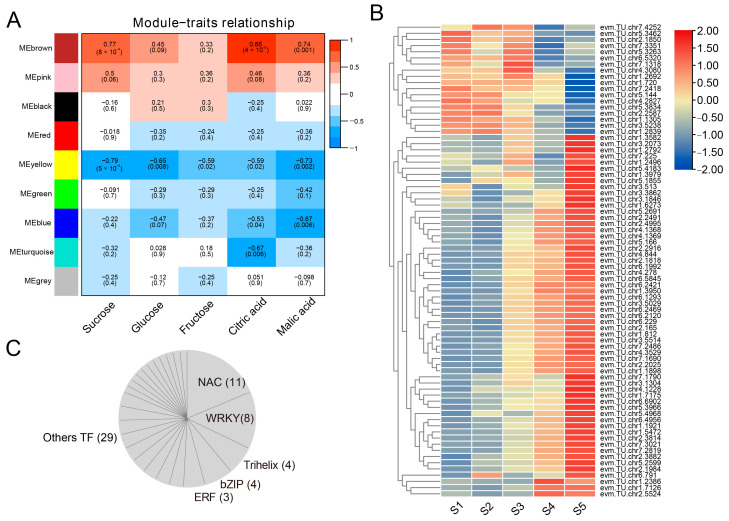
Weighted gene co-expression network analysis (WGCNA) of transcription factors. (**A**) Relationships of gene modules to sugars and organic acids. (**B**) Expression profiles of transcription factors with FPKM values > 10 in the brown module. (**C**) Classification of transcription factors that were upregulated during *Rosa roxburghii* fruit development.

To investigate the key transcription factors involved in regulating sugar and organic acid synthesis in *Rosa roxburghii*, we first identified a total of 2496 high confidence transcription factors (Table S4). Additionally, WGCNA was used to construct a network of key transcription factors using sugar and organic acid contents in *Rosa roxburghii* fruits as the phenotypic data. As shown in Figure 5A, all transcription factors were divided into nine modules. The brown module was significantly positively correlated with sucrose, citric acid and malic acid, with r2 values of 0.77, 0.86 and 0.74, respectively. Interestingly, no modules were significantly correlated with glucose or fructose. We also found that the yellow, green and blue modules were significantly negatively correlated with one or more different sugars or organic acids. These results suggest that transcription factors are closely related to the metabolism of *Rosa roxburghii* sugars and organic acids.

**Figure 6:**
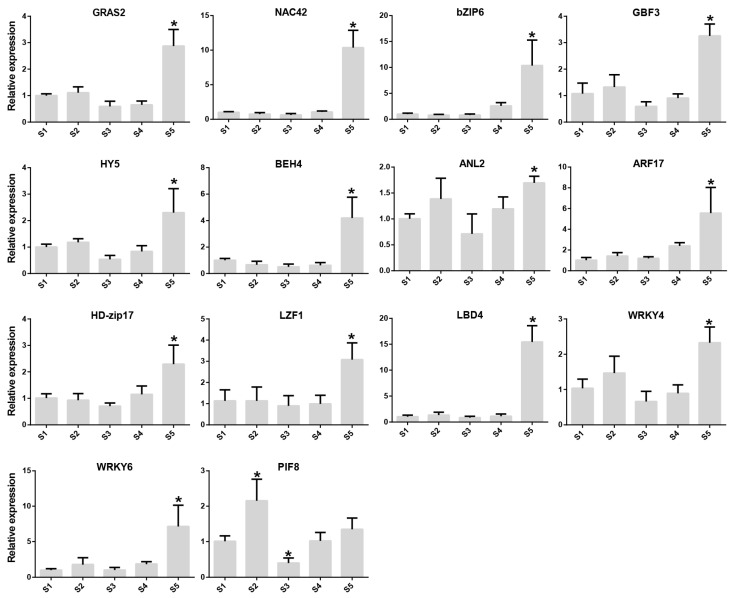
Relative expression of 14 transcription factors involved in sugar and organic acid metabolism. Asterisk indicates significant difference (LSD, *p* < 0.05).

There was a total of 132 transcription factors included in the brown module (Table S5). After filtering to remove low-abundance transcripts (FPKM value > 10), 78 genes were retained and used to construct a network (Figure 5B). Within this network, a total of 59 transcription factors were upregulated over the course of *Rosa roxburghii* fruit development. These 59 transcription factors included NAC (11), WRKY (4), trihelix (4), bZIP (4), ERF (3) and others (Figure 5C). The gene with the largest fold difference in expression between S1 and S5 stages was evm.TU.chr6.2120 (LBD4, 202.87-fold), followed by evm.TU.chr3.5029 (HD-zip, 15.45-fold), evm.TU.chr1.1921 (NAC42, 9.69-fold) and evm.TU.chr1.5472 (ZIP6, 5.04-fold) (Table S6). We selected 14 genes (fold change ≥2) for qPCR analysis, including RrANL2, RrARF17, RrBEH4, RrbZIP6, RrGBF3, RrGRAS2, RrHD-zip17, RrHY5, RrLBD4, RrLZF1, RrNAC42, RrPIF8, RrWRKY3 and RrWRKY6. This analysis revealed expression changes that were similar to the RNA-seq data for all transcription factors, except PIF8, with detectible gene expression for all genes at S5 (Figure 6). Taken together, these results indicate that a large number of different transcription factors may be involved in regulating sugar and organic acid metabolism of *Rosa roxburghii* fruit.

### 2.5. Identification of Transcription Factors Involved in the Metabolism of Rosa roxburghii Sugars and Organic Acids

**Figure 7:**
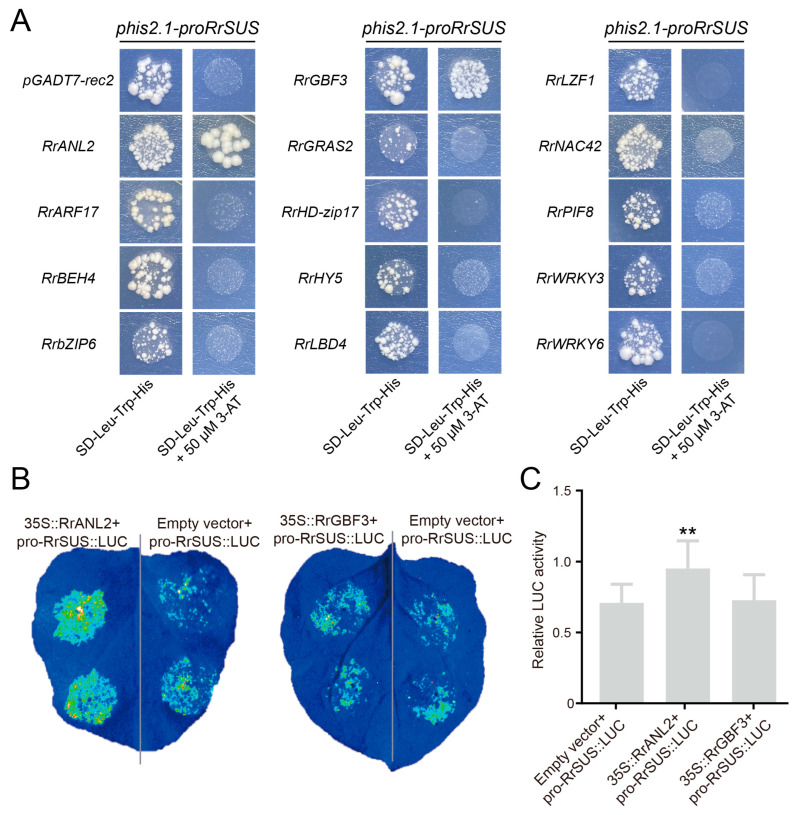
Validation of transcription factor activity. (**A**) Validation of the interaction of 14 transcription factors with the RrSUS promoter. The 3-AT concentration was 50 µM. (**B**,**C**) Transient expression in tobacco leaves to verify the LUC activity of transcription factors. Asterisk indicates significant difference (LSD, *p* < 0.01).

*RrSUS3*, *RrME1* and *RrCS2* are key genes regulating sucrose, malic acid and citric acid synthesis in *Rosa roxburghii* fruit. We therefore investigated the interactions between the above 14 transcription factors and the promoters of these three genes. Yeast one-hybrid experiments revealed that no transcription factors bound to the promoters of evm.TU.chr1.7029 (*RrME1*) and evm.TU.chr1.2040 (*RrCS2*), whereas *RrANL2* and *RrGBF3* bound to the promoter of evm.TU.chr4.1713 (*RrSUS3*) (Figure 7A). 

To further understand the interactions between transcription factors (*RrANL2* and *RrGBF3*) and *RrSUS*, we performed dual luciferase assays. This analysis showed that *RrANL2* could enhance the fluorescence signal of pro-RrSUS::LUC, whereas *RrGBF3* had no effect on the fluorescence signal of pro-RrSUS::LUC (Figure 7B,C). These results suggest that *RrANL2* impacts sucrose accumulation in *Rosa roxburghii* fruit by regulating the expression of *RrSUS3*.

## 3. Discussion

Fruit development is impacted by various environmental factors, genes and hormones, and the resulting sugars and organic acids produced during this process have a significant impact on fruit quality. In this study, transcriptomic and metabolomic data were used to construct the first networks that control sugars and organic acids in *Rosa roxburghii* fruit.

### 3.1. Accumulation Patterns of Sugars and Organic Acids in Rosa roxburghii

Organic acids usually accumulate significantly in the early stages of fruit development but decrease as development progresses. These changes are thought to be largely due to the increased synthesis of sugars during the later stages of fruit development [17]. In this study, we found that the main organic acids in *Rosa roxburghii* fruit were malic acid and citric acid, whose levels increased continuously during fruit development and peaked at fruit ripening. This was consistent with the accumulation pattern of organic acids in apples, peaches, plums, loquats and watermelons [18,19,20]. In addition, we found that sucrose, glucose and fructose all increased during *Rosa roxburghii* fruit ripening, but each had a distinct accumulation pattern. Glucose and fructose contents were found to be very high at the beginning of fruit development, but their content suddenly decreased midway through development, before accumulating again at fruit ripening. This pattern of changes was different from other more well-studied fruits, such as jujube, pear, watermelon and others, making it important to better understand the regulatory networks underlying changes in sugars and organic acids during *Rosa roxburghii* fruit development [19,21,22]. 

### 3.2. Regulatory Network of Rosa roxburghii Sugars and Organic Acids

The synthesis and catabolism of sugars and organic acids have significant impacts on the resulting fruit quality, and they are often regulated by multiple metabolic pathways. Previous studies have shown that the expression of a single gene does not have a significant effect on the overall sugar and organic acid accumulation in fruit. For example, studies have shown that perturbations in the sugar transporter protein *CiTST2* in watermelon [5], the sugar transporter protein PpTST1 in peach [21], the sugar transporter protein *CsPH8* in citrus fruit [23] and the ALMT gene family [13,24,25] often have minor effects on total sugar and organic acid content. We therefore constructed the metabolic pathways of sugar and organic acids in *Rosa roxburghii* and identified several genes that were significantly expressed at fruit ripening. Analysis of these pathways showed that genes involved in sugar and organic acid metabolism had highly similar expression trends, suggesting that the sugars and organic acids are regulated by complex networks.

Numerous studies have shown that transcription factors play important roles in the accumulation of sugars and organic acids in fruits. For example, the strawberry NAC transcription factor *FaRIF* promotes sugar accumulation in fruit [26], the grape transcription factor *WRKY22* regulates sucrose non-fermenting-1-related protein kinase 1 to promote sugar accumulation [27], and the transcription factor *AREB2* in apple acts on sugar transporter proteins and amylases to promote sugar accumulation [28]. Additionally, tomato *SlAREB1* promotes the accumulation of organic acids [29], strawberry *FaMYB5* participates in citric acid metabolism [30] and *CitERF13* regulates *CitVHA* to alter citric acid accumulation in citrus fruit [23]. We therefore further divided all transcription factors in the *Rosa roxburghii* genome into 10 modules by WGCNA, and found that the brown module, which contained 59 significantly expressed transcription factors, was significantly associated with sucrose, citric acid and malic acid.

### 3.3. Transcription Factor RrANL2 Regulates the Involvement of RrSUS3 in Sucrose Metabolism

We analyzed the expression of 14 transcription factors during *Rosa roxburghii* fruit development and found that most were consistent with the expression patterns of genes related to sucrose, malic acid and citric acid synthesis. Furthermore, expression of these transcription factors was consistent with changes in metabolite content, indicating the importance of transcription factors in sugar and organic acid metabolism in *Rosa roxburghii*. Subsequent yeast one-hybrid assays and dual luciferase assays showed that the transcription factor *RrANL2* can bind to the promoter of RrSUS3 to promote its expression, impacting sucrose levels and potentially affecting yield, fiber quality, fruit quality and growth rate [31,32,33,34,35]. Additionally, *ANL2* (ANTHOCYANINLESS2) belongs to the HD-ZIP IV subfamily [35] and functions in the development of plant root epidermal cells and the accumulation of anthocyanin [36,37,38]. *ANL2* has been shown to be involved in sucrose metabolism in plants, where it impacts the level of the sucrose transporter protein *AtSUC1* in Arabidopsis thaliana [39]. Additionally, sucrose acts as a molecular signal to regulate anthocyanin accumulation [38]. In this study, we found that *RrANL2* was an upstream regulator of *RrSUS3*, which provides new insights into the regulation of sugar metabolism in *Rosa roxburghii* fruit.

In this study, we integrated transcriptomic and metabolomic techniques to investigate the accumulation patterns of sugars and organic acids during *Rosa roxburghii* fruit development. During this process, genes associated with sugar and organic acid pathways were differentially expressed, with biosynthetic genes primarily increasing in expression during fruit development. Additionally, numerous transcription factors that may control sugar and organic acid metabolism in *Rosa roxburghii* were identified by WGCNA. Moreover, the transcription factor *RrANL2* was found to regulate the expression of RrSUS3, a key gene in sucrose synthesis. Taken together, these results partially elucidate the metabolism of sugars and organic acids in *Rosa roxburghii* and provide a biological basis for understanding their accumulation in *Rosa roxburghii* fruit. 

## 4. Materials and Methods

### 4.1. Plant Materials

The *Rosa roxburghii* material used in this study was collected from the Baima Experimental Base of Nanjing Agricultural University, Jiangsu Province, China. Three biological replicates were collected for each sample 10, 40, 70, 100 and 130 days after flowering and named S1, S2, S3, S4 and S5. Samples were frozen in liquid nitrogen after collection and stored in a −80 °C freezer for later use.

### 4.2. Extraction and Analysis of Inclusions

Inclusions of samples from the five fruit stages were dissolved in 70% methanol and left overnight at 4 °C. After centrifugation at 10,000 rpm for 10 min, the supernatant was analyzed using a UPLC-ESI-MS/MS system (UPLC–SHIMADZU Nexera X2; MS–Applied Biosystems 4500 Q TRAP). Metabolites were considered differentially accumulated when they had an absolute Log2FC (fold change) ≥ 1 with a VIP ≥ 1. Metabolites were annotated and examined for enrichment using the KEGG compound database (http://www.kegg.jp/kegg/compound/, accessed on 11 February 2022) and KEGG pathway database (http://www.kegg.jp/kegg/pathway.html, accessed on 11 February 2022). 

### 4.3. RNA-Seq Library Construction and Data Analysis

Total RNA was extracted from five stages of fruit using an RNAprep Pure Plant Plus Kit (Tiangen, Beijing, China). RNA concentration and purity were measured using a NanoDrop 2000 spectrophotometer (Thermo Fisher Scientific, Wilmington, DE, USA), and RNA integrity was assessed using an RNA Nano 6000 Assay Kit from the Agilent Bioanalyzer 2100 system (Agilent Technologies, Santa Clara, CA, USA). After quality assessment, 1 µg of RNA was utilized for RNA-seq library construction. Paired-end RNA-seq reads were generated using the Illumina NovaSeq 6000 platform. Raw RNA-seq data were then filtered with fastp [40] and mapped against the *Rosa roxburghii* genome by HISAT2 with default parameters [41]. We calculated the fragments per kilobase of transcript per million fragments mapped (FPKM) metric from featureCounts for each gene and identified differentially expressed genes with the DEseq2 R package, using a cutoff of adjusted *p*-value < 0.05. We then performed KEGG enrichment analysis of differentially expressed genes with clusterProfiler. 

### 4.4. Identification of Key Transcription Factors by WGCNA

To understand the effects of transcription factors on sugars and organic acids during *Rosa roxburghii* fruit development, we identified transcription factors in the *Rosa roxburghii* genome by BLASTP [42] using all transcription factors of Arabidopsis thaliana as queries. The identified transcription factors were further analyzed via hmmscan to determine their conserved structural domains [43]. Additionally, we used WGCNA to construct a gene regulatory network for sugars and organic acids during *Rosa roxburghii* fruit development [44]. Using glucose, fructose, sucrose, malic acid and citric acid as phenotypic data, we normalized and iteratively examined expression data for all transcription factors. 

### 4.5. Total RNA Extraction and qRT-PCR Analysis

Total RNA was extracted from five stages of *Rosa roxburghii* fruit using a Foregene Plant Total RNA Extraction kit (Chengdu, China). RNA was subsequently reverse-transcribed into cDNA by the PrimeScript RT Reagent Kit (TaKaRa, Beijing, China). The relative expression patterns of target genes during *Rosa roxburghii* fruit development were analyzed with the Hieff UNICON Universal Blue qPCR SYBR Green Master Mix (YEASEN, Shanghai, China). We utilized the expression of *Rosa roxburghii* RrUBQ as a reference gene for calculating relative expression by the 2^−∆Ct^ method and determined statistical significance using SPSS22. All primers used in this analysis are listed in Table S1.

### 4.6. Yeast One-Hybrid and Dual Luciferase Assays

We obtained total RNA and genomic DNA from *Rosa roxburghii* fruit using the Foregene Total Plant RNA Extraction Kit and Foregene Plant DNA Extraction Kit, respectively. cDNA was obtained by reverse transcription of RNA using the PrimeScript RT Reagent Kit (TaKaRa). The transcription factor sequences were amplified by PrimeSTAR HS DNA Polymerase (TaKaRa) and ligated into pGADT7 and PJX001 vectors. Additionally, the promoter sequence of each target gene was amplified from *Rosa roxburghii* genomic DNA and ligated into pHIS2.1 and pGreenII 0800-LUC vectors. All primers used in this analysis are listed in Table S1.

Yeast one-hybrid assays were used to determine the binding relationships between transcription factors and promoters. The pHIS2.1 vector carrying the transcription factor pGADT7 vector and the pHIS2.1 vector carrying the promoter sequence were transformed into the Y187 yeast strain by a modified lithium acetate method. The yeast strain was subsequently plated on SD-Leu-Trp solid agar medium (Coolaber, BeiJing, China) and incubated at 30 °C. After 3 days, single colonies of yeast were picked, suspended in ddH_2_O and their concentration was adjusted to an OD600 of 0.05. Next, 5 µL of the yeast strain suspension was aspirated and transferred to SD/-Leu/-Trp/-His liquid medium (Coolaber, BeiJing, China) containing different concentrations of 3-AT (0, 25, 50, 75 and 100 µM) and grown for 4 days at 30 °C.

The regulatory relationship between transcription factors and promoters was further verified using dual luciferase assays. PJX001 vectors carrying transcription factors and pGreenII 0800-LUC vectors carrying promoter sequences were transformed into the Agrobacterium tumefaciens GV3101 receptor. The different combinations of transcription factors and promoters were then injected into tobacco leaves in a 9:1 ratio. After 3 days, tobacco leaves were assessed for LUC activity to determine the interactions between different promoters and transcription factors.

## Data Availability

All sequencing data, including that of the genome and transcriptome, can be found in the China National Center for Bioinformation database (PRJCA014460).

## Supplementary Materials

The following supporting information can be downloaded at: https://www.mdpi.com/article/10.3390/plants12173036/s1, Table S1. Primers used in this study; Table S2. Sugar and organic acid contents of *Rosa roxburghii* during fruit ripening; Table S3. FPKM values of genes related to sugar and organic acid metabolism; Table S4. Transcription factor identification of *Rosa roxburghii*; Table S5. The FPKM value of transcription factor in MEbrown module; Table S6. Transcription factors expression fold change of S5 vs. S1.

## References

[1] Ackermann J., Fischer M., Amado R. (1992). Changes in sugars, acids, and amino acids during ripening and storage of apples (cv. Glockenapfel). J. Agric. Food Chem..

[2] Kroger M., Meister K., Kava R. (2006). Low-calorie Sweeteners and Other Sugar Substitutes: A Review of the Safety Issues. Compr. Rev. Food Saf..

[3] Pangborn R. (2006). Relative Taste Intensities of Selected Sugars and Organic Acids. J. Food Sci..

[4] Shiraishi M., Fujishima H., Chijiwa H. (2010). Evaluation of table grape genetic resources for sugar, organic acid, and amino acid composition of berries. Euphytica.

[5] Ren Y., Guo S., Zhang J., He H., Sun H., Tian S., Gong G., Zhang H., Levi A., Tadmor Y. (2018). A Tonoplast Sugar Transporter Underlies a Sugar Accumulation QTL in Watermelon. Plant Physiol..

[6] Lingle S.E., Dunlap J.R. (1987). Sucrose Metabolism in Netted Muskmelon Fruit during Development. Plant Physiol..

[7] Wu J., Gao H., Zhao L., Liao X., Chen F., Wang Z., Hu X. (2007). Chemical compositional characterization of some apple cultivars. Food Chem..

[8] Zhang H.P., Wu J.Y., Qin G.H., Yao G.F., Qi K.J., Wang L.F., Zhang S.L. (2014). The role of sucrose-metabolizing enzymes in pear fruit that differ in sucrose accumulation. Acta Physiol. Plant..

[9] Ruan Y. (2014). Sucrose Metabolism: Gateway to Diverse Carbon Use and Sugar Signaling. Annu. Rev. Plant Biol..

[10] Rolland F., Baena-Gonzalez E., Sheen J. (2006). Sugar Sensing and Signaling in Plants: Conserved and Novel Mechanisms. Annu. Rev. Plant Biol..

[11] Montero T.M., Mollá E.M., Esteban R.M., López-Andréu F.J. (1996). Quality attributes of strawberry during ripening. Sci. Hortic.-Amst..

[12] Giné Bordonaba J., Terry L.A. (2010). Manipulating the taste-related composition of strawberry fruits (Fragaria×ananassa) from different cultivars using deficit irrigation. Food Chem..

[13] Etienne A., Génard M., Lobit P., Mbeguié-A-Mbéguié D., Bugaud C. (2013). What controls fleshy fruit acidity? A review of malate and citrate accumulation in fruit cells. J. Exp. Bot..

[14] Zhang Y., Fernie A.R. (2018). On the role of the tricarboxylic acid cycle in plant productivity. J. Integr. Plant Biol..

[15] Li H., Fang W., Wang Z., Chen Y. (2022). Physicochemical, biological properties, and flavour profile of Rosa roxburghii Tratt, Pyracantha fortuneana, and Rosa laevigata Michx fruits: A comprehensive review. Food Chem..

[16] Wang L., Lv M., An J., Fan X., Dong M., Zhang S., Wang J., Wang Y., Cai Z., Fu Y. (2021). Botanical characteristics, phytochemistry and related biological activities of Rosa roxburghii Tratt fruit, and its potential use in functional foods: A review. Food Funct..

[17] Lombardo V.A., Osorio S., Borsani J., Lauxmann M.A., Bustamante C.A., Budde C.O., Andreo C.S., Lara M.V., Fernie A.R., Drincovich M.F. (2011). Metabolic Profiling during Peach Fruit Development and Ripening Reveals the Metabolic Networks That Underpin Each Developmental Stage. Plant Physiol..

[18] Famiani F., Battistelli A., Moscatello S., Cruzcastillo J.G., Walker R.P. (2015). The organic acids that are accumulated in the flesh of fruits: Occurrence, metabolism and factors affecting their contents—A review. Rev. Chapingo Ser. Hortic..

[19] Umer M.J., Bin Safdar L., Gebremeskel H., Zhao S., Yuan P., Zhu H., Kaseb M.O., Anees M., Lu X., He N. (2020). Identification of key gene networks controlling organic acid and sugar metabolism during watermelon fruit development by integrating metabolic phenotypes and gene expression profiles. Hortic. Res..

[20] Yao Y.X., Ming L., Liu Z., You C.X., Wang D.M., Zhai H., Hao Y.J. (2009). Molecular cloning of three malic acid related genes MdPEPC, MdVHA-A, MdcyME and their expression analysis in apple fruits. Sci. Hortic..

[21] Peng Q., Wang L., Ogutu C., Liu J., Liu L., Mollah M., Han Y. (2020). Functional Analysis Reveals the Regulatory Role of PpTST1 Encoding Tonoplast Sugar Transporter in Sugar Accumulation of Peach Fruit. Int. J. Mol. Sci..

[22] Zhang C., Bian Y., Hou S., Li X. (2018). Sugar transport played a more important role than sugar biosynthesis in fruit sugar accumulation during Chinese jujube domestication. Planta.

[23] Li S., Yin X., Xie X., Allan A.C., Ge H., Shen S., Chen K. (2016). The Citrus transcription factor, CitERF13, regulates citric acid accumulation via a protein-protein interaction with the vacuolar proton pump, CitVHA-c4. Sci. Rep..

[24] Angeli A., Baetz U., Francisco R., Zhang J., Chaves M.M., Regalado A. (2013). The vacuolar channel VvALMT9 mediates malate and tartrate accumulation in berries of Vitis vinifera. Planta.

[25] Etienne C., Rothan C., Moing A., Plomion C., Bodénès C., Svanella-Dumas L., Cosson P., Pronier V., Monet R., Dirlewanger E. (2002). Candidate genes and QTLs for sugar and organic acid content in peach [*Prunus persica* (L.) Batsch]. Theor. Appl. Genet..

[26] Martín-Pizarro C., Vallarino J.G., Osorio S., Meco V., Urrutia M., Pillet J., Casañal A., Merchante C., Amaya I., Willmitzer L. (2021). The NAC transcription factor FaRIF controls fruit ripening in strawberry. Plant Cell.

[27] Huang T., Yu D., Wang X. (2021). VvWRKY22 transcription factor interacts with VvSnRK1.1/VvSnRK1.2 and regulates sugar accumulation in grape. Biochem. Bioph. Res. Commun..

[28] Ma Q.J., Sun M.H., Lu J., Liu Y.J., Hu D.G., Hao Y.J. (2017). Transcription Factor AREB2 Is Involved in Soluble Sugar Accumulation by Activating Sugar Transporter and Amylase Genes. Plant Physiol..

[29] Bastías A., López-Climent M., Valcárcel M., Rosello S., Gómez-Cadenas A., Casaretto J.A. (2011). Modulation of organic acids and sugar content in tomato fruits by an abscisic acid-regulated transcription factor. Physiol. Plant..

[30] Liu Y., Zhu L., Yang M., Xie X., Sun P., Fang C., Zhao J. (2022). R2R3-MYB transcription factor FaMYB5 is involved in citric acid metabolism in strawberry fruits. J. Plant Physiol..

[31] Barrero-Sicilia C., Hernando-Amado S., González-Melendi P., Carbonero P. (2011). Structure, expression profile and subcellular localisation of four different sucrose synthase genes from barley. Planta.

[32] Poovaiah C.R., Mazarei M., Decker S.R., Turner G.B., Sykes R.W., Davis M.F., Stewart C.N. (2015). Transgenic switchgrass (Panicum virgatum L.) biomass is increased by overexpression of switchgrass sucrose synthase (PvSUS1). Biotechnol. J..

[33] Qazi H.A., Paranjpe S., Bhargava S. (2012). Stem sugar accumulation in sweet sorghum—Activity and expression of sucrose metabolizing enzymes and sucrose transporters. J. Plant Physiol..

[34] Ruan Y. (2012). Signaling Role of Sucrose Metabolism in Development. Mol. Plant.

[35] Nakamura M., Katsumata H., Abe M., Yabe N., Komeda Y., Yamamoto K.T., Takahashi T. (2006). Characterization of the Class IV Homeodomain-Leucine Zipper Gene Family in Arabidopsis. Plant Physiol..

[36] Kubo H., Hayashi K. (2011). Characterization of root cells of anl2 mutant in Arabidopsis thaliana. Plant Sci..

[37] Nadakuduti S.S., Pollard M., Kosma D.K., Allen C., Ohlrogge J.B., Barry C.S. (2012). Pleiotropic Phenotypes of the sticky peel Mutant Provide New Insight into the Role of CUTIN DEFICIENT2 in Epidermal Cell Function in Tomato. Plant Physiol..

[38] Solfanelli C., Poggi A., Loreti E., Alpi A., Perata P. (2006). Sucrose-Specific Induction of the Anthocyanin Biosynthetic Pathway in Arabidopsis. Plant Physiol..

[39] Sivitz A.B., Ward R.J.M. (2008). Arabidopsis sucrose transporter AtSUC1 is important for pollen germination and sucrose-induced anthocyanin accumulation. Plant Physiol..

[40] Chen S., Zhou Y., Chen Y., Gu J. (2018). fastp: An ultra-fast all-in-one FASTQ preprocessor. Bioinformatics.

[41] Kim D., Langmead B., Salzberg S.L. (2015). HISAT: A fast spliced aligner with low memory requirements. Nat. Methods.

[42] Altschul S.F. (2012). Basic local alignment search tool (BLAST). J. Mol. Biol..

[43] Johnson L.S., Eddy S.R., Portugaly E. (2010). Hidden Markov model speed heuristic and iterative HMM search procedure. BMC Bioinform..

[44] Langfelder P., Horvath S. (2008). WGCNA: An R package for weighted correlation network analysis. BMC Bioinform..

